# Crystal structure and Hirshfeld surface analysis of a halogen bond between 2-(allyl­thio)­pyridine and 1,2,4,5-tetra­fluoro-3,6-di­iodo­benzene

**DOI:** 10.1107/S2056989024005693

**Published:** 2024-06-21

**Authors:** Robin Risken, Tobias Schrimpf, Franziska Dorothea Klotz, Carsten Strohmann

**Affiliations:** a Inorganic Chemistry, TU Dortmund University, Otto-Hahn Str. 6, 44227 Dortmund, Germany; Katholieke Universiteit Leuven, Belgium

**Keywords:** crystal structure, halogen bond, Hirshfeld surface analysis

## Abstract

The crystal structure of the title 2:1 mol­ecular complex between 2-(allyl­thio)­pyridine and 1,2,4,5-tetra­fluoro-3,6-di­iodo­benzene, C_6_F_4_I_2_·2C_8_H_9_NS, at 100 K has been determined in the monoclinic space group *P*2_1_/*c*. The most noteworthy characteristic of the complex is the halogen bond between iodine and the pyridine ring with a short N⋯I contact [2.8628 (12) Å]. The Hirshfeld surface analysis shows that the hydrogen⋯hydrogen contacts dominate the crystal packing with a contribution of 32.1%.

## Abstract 2

The crystal structure of the mol­ecular complex between 2-(allyl­thio)­pyridine and 1,2,4,5-tetra­fluoro-3,6-di­iodo­benzene, which exhibits an N⋯I halogen bond, has been determined at 100 K.

## 1. Chemical context

**Figure 1:**
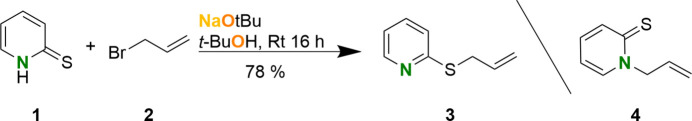
Synthesis of 2-(allyl­thio)­pyridine **3** or 1-allyl­pyridine-2(1*H*)-thione **4**.

Earlier research investigated the deprotonation of allylic silicon compounds with organolithium reagents (Strohmann *et al.*, 2006 ▸). In this work, 2-(allyl­thio)­pyridine was synthesized to compare the chemical behavior to similar systems. The chosen synthetic route was adapted from the literature (Baudin *et al.*, 1993 ▸) and could lead to two similar products (Fig. 1 ▸) that would be hard to distinguish based on ^1^H- and ^13^C-NMR alone. Since the 2-(allyl­thio)­pyridine did not crystallize and was quite impure, 1,2,4,5,-tetra­fluoro-3,6-di­iodo­benzene was added, which led to a two-component co-crystal referred to as complex **5**. Halogen bonds between an aromatic iodine compound and a nitro­gen compound can vary in their bond strength, length and angle (Otte *et al.*, 2023 ▸). Since only the desired compound formed a halogen bond, the product could be separated from the impurities by simply isolating the co-crystals. Afterwards the 2-(allyl­thio)­pyridine was reobtained by column chromatography.

## 2. Structural commentary

**Figure 2:**
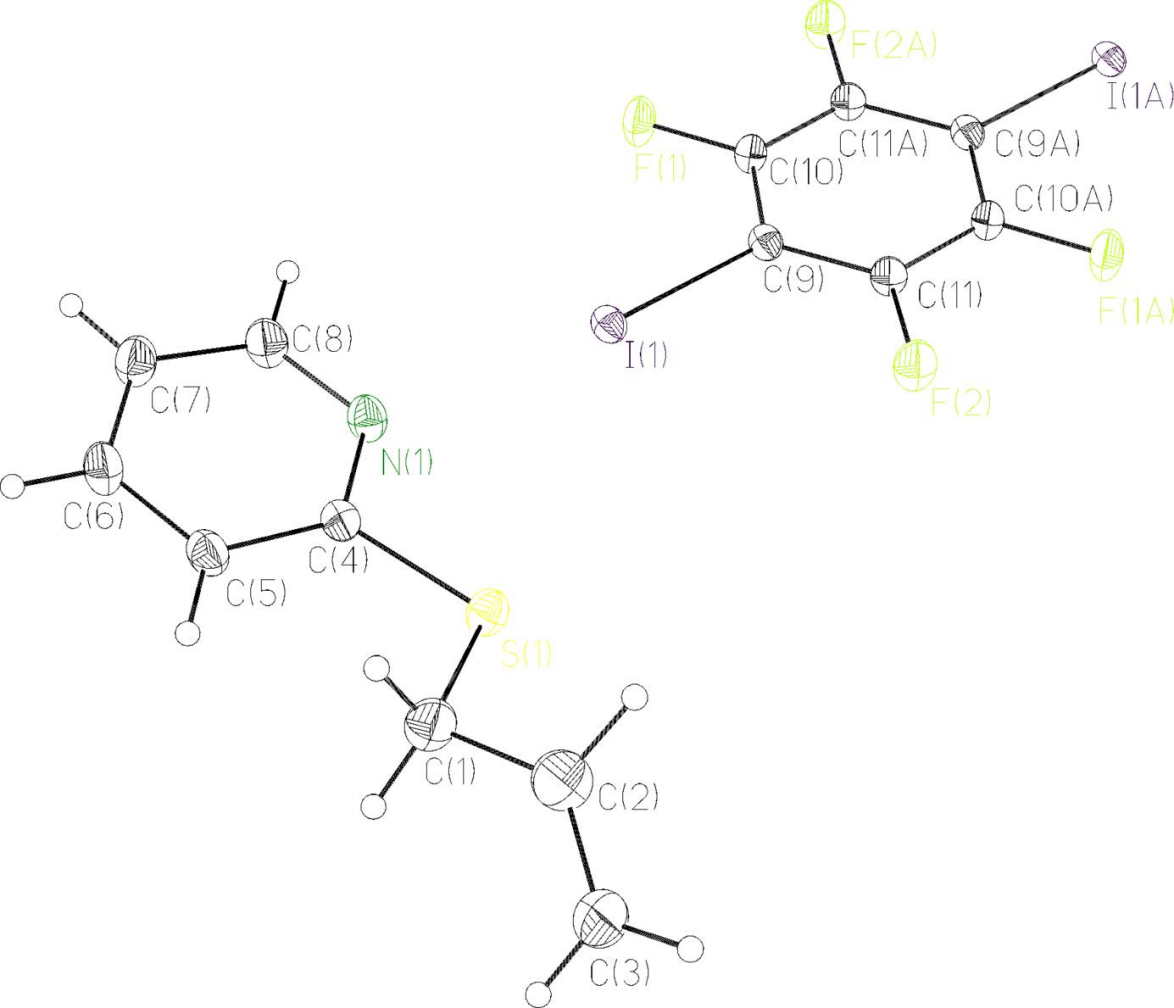
The mol­ecular structure of the title compound **5**, showing the atom labeling and displacement ellipsoids drawn at the 50% probability level.

Complex **5** crystallized from heptane at 193.15 K as colorless plates in the monoclinic space group *P*2_1_/*c.* The asymmetric unit consists of one mol­ecule of **3** and half a mol­ecule of 1,2,4,5,-tetra­fluoro-3,6-di­iodo­benzene. The second half is generated by inversion symmetry (Fig. 2 ▸; symmetry operation −*x*, −*y*, 1 − *z*). The formula unit of the title compound consists of two mol­ecules 2-(allyl­thio)­pyridine and one mol­ecule 1,2,4,5,-tetra­fluoro-3,6-di­iodo­benzene, which lies on an inversion center.
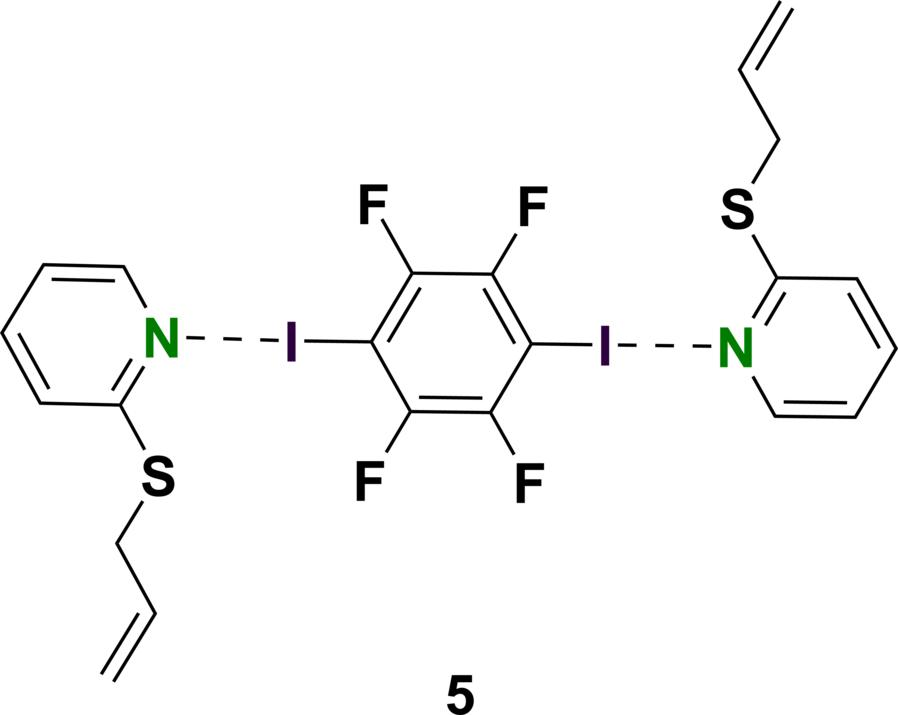


The complex consists of multiple functional groups: an allyl group, a thio­ether, a pyridine and a perfluorinated di­iodo­benzene. The C1—C2 bond length [1.492 (2) Å] is longer than the C2—C3 bond length [1.316 (3) Å], which is explained by the double bond between C2 and C3. These lengths coincide with the C—C single bond length of 1.54 Å in ethane and the C=C double bond length of 1.33 Å in ethene (Lide, 2005 ▸). The C4—S1—C1 bond angle [102.91 (7)°] is a little bit larger than the C—S—C angle in di­methyl­sulfide [99.2 (1)°; Mitzel & Losehand, 2004 ▸]. The difference in bond angles might be explained by the larger pyridine substituent, of which C4 is a part. The bond lengths in the pyridine ring [C4—C5: 1.3976 (18) Å, C5—C6: 1.391 (2) Å, C6—C7: 1.387 (2) Å, C7—C8: 1.384 (2) Å, C8—N1: 1.3454 (18) Å and N1—C4: 1.3422 (17) Å] are not significantly longer compared to the bond lengths in pyridine (Lide, 2005 ▸). The bond angles of the pyridine moiety vary around 118°, which is typical for pyridine. The largest deviations from planarity of the pyridine (r.m.s. deviation 0.010 Å) are observed for C7 [–0.0124 (12) Å] and C4 [–0.0140 (9) Å]. The angle between the normal of the pyridine plane (N1,C4–C8) and the double bond between C2 and C3 is 115.35 (13)°.

The bond lengths and angles of 1,2,4,5,-tetra­fluoro-3,6-di­iodo­benzene are consistent with those present in the Cambridge Structural Database. The benzene ring makes a dihedral angle of 12.88 (5)° with the pyridine ring.

## 3. Supra­molecular features

**Figure 3:**
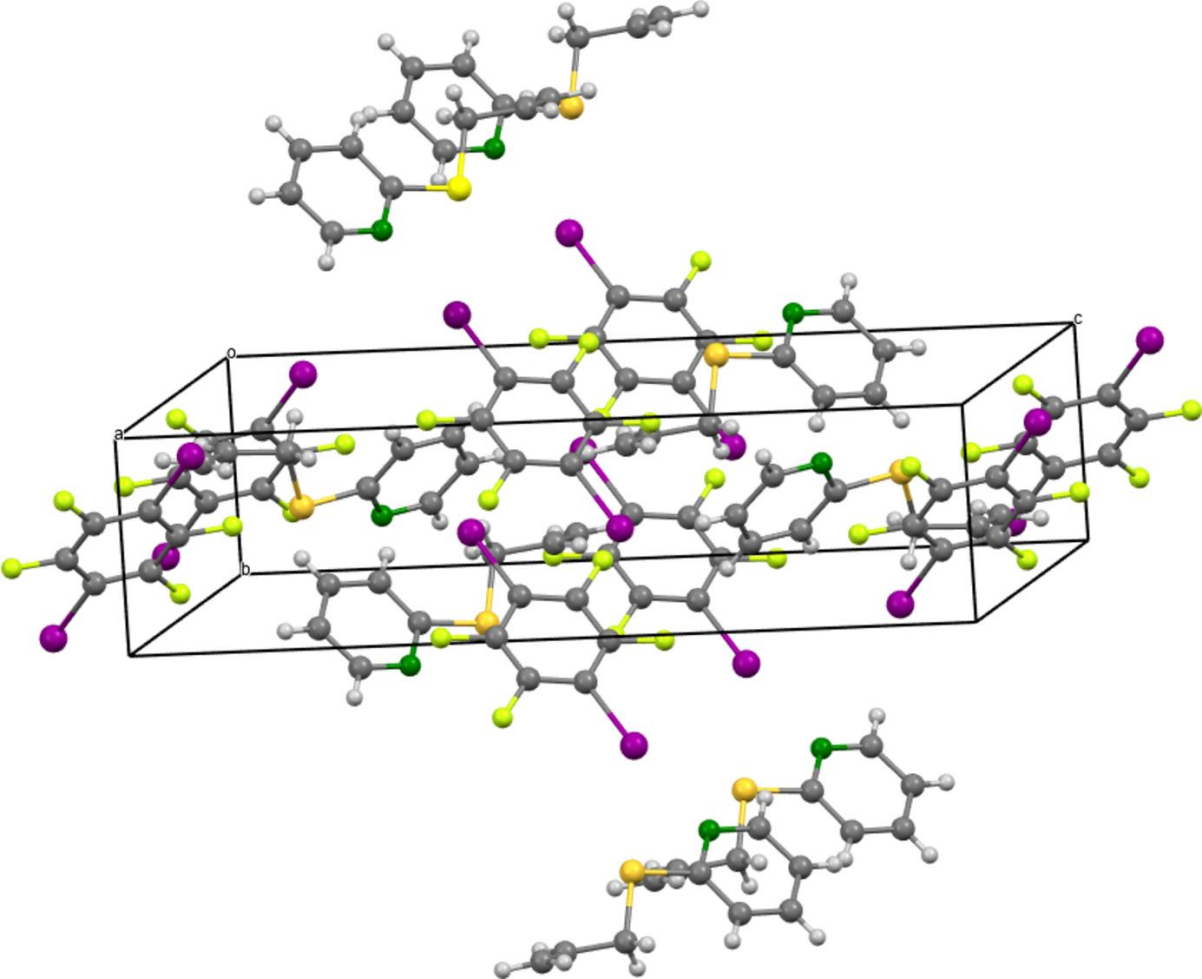
A view of the packing of **5**.

**Figure 4:**
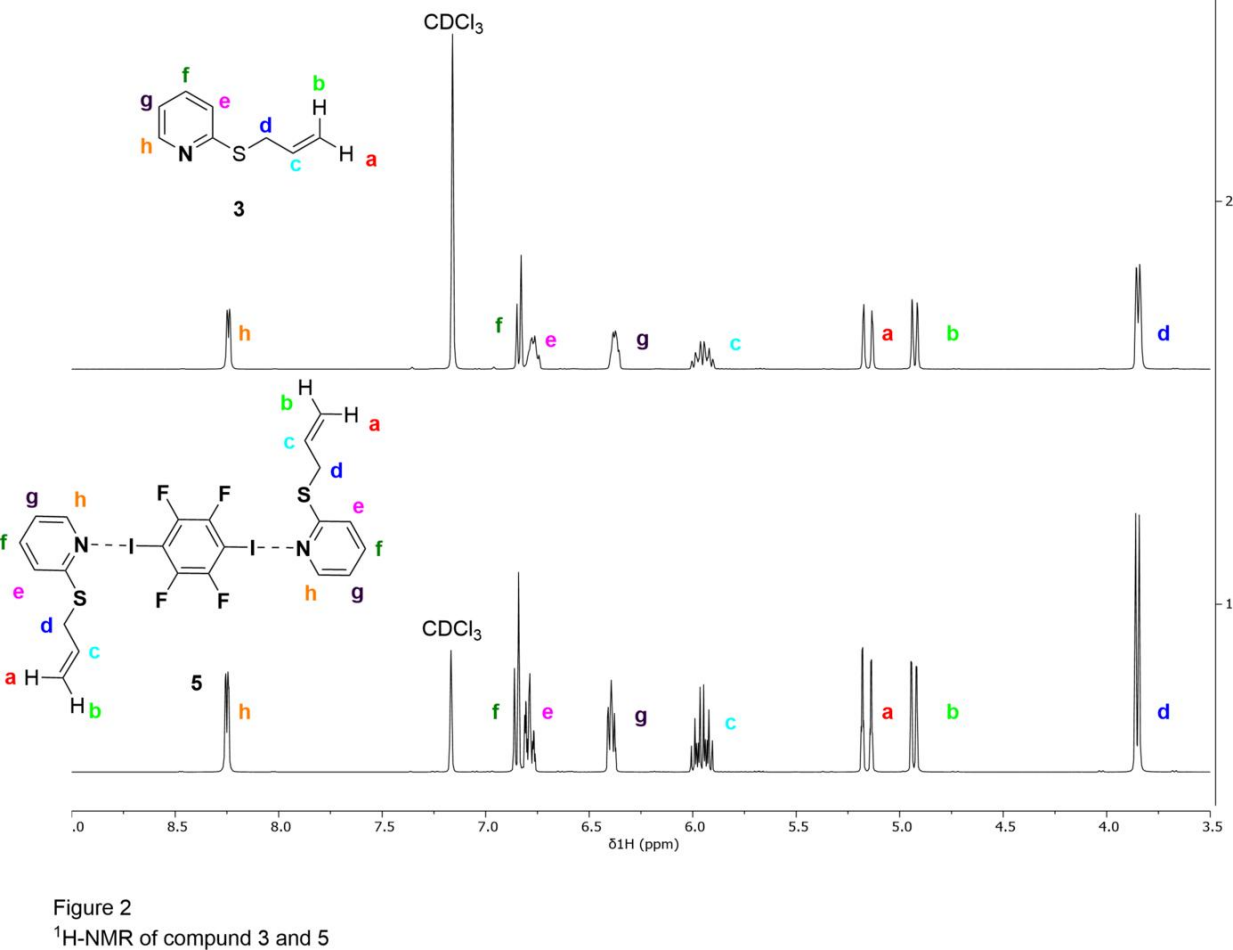
^1^H-NMR spectra (400 MHz) of compounds **3** and **5** in CDCl_3_.

Fig. 3 ▸ shows the packing of the complex. The most important supra­molecular feature is the close contact between N1 and I1 with a coordination distance of 2.8628 (12) Å, which is shorter than the sum of the van der Waals radii of 3.73 Å (Nyburg & Faerman, 1985 ▸). The strength of a halogen bond is determined by the bond length and the bond angle. Strong halogen bonds are expected to have a bond length in the region of 2.781 (2) Å (Otte *et al.*, 2023 ▸), which is shorter than the distance observed for **5**. The N1—I1—C9 bond angle is 173.90 (4)°, which is slightly less than a theoretical ideal angle of 180°. In conclusion, the here presented structure shows a medium-strength halogen bond between N1 and I1. Further evidence for this is the small difference in ^1^H-NMR chemical shifts between compounds **3** and **5** (Fig. 4 ▸). Theoretically, the sulfur present here could also form a halogen bond with the iodine, but this behavior is not observed.

**Figure 5:**
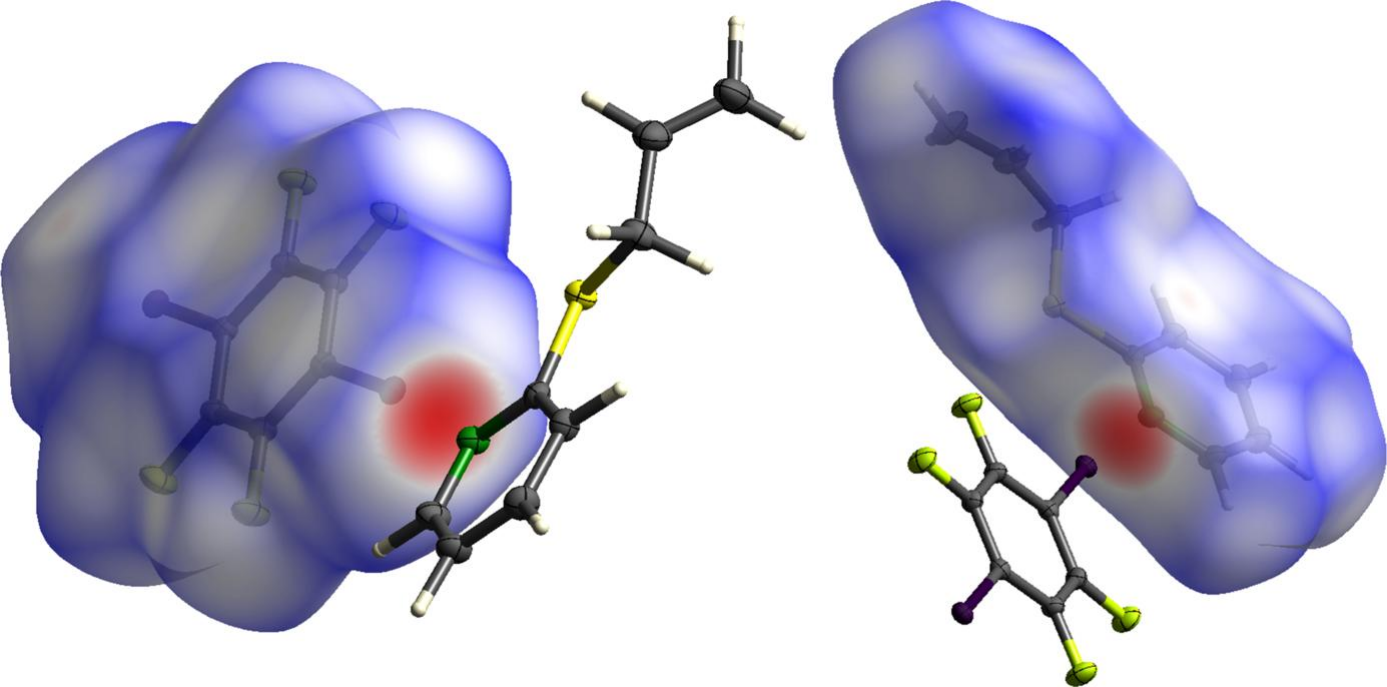
Three-dimensional Hirshfeld surface of **5** mapped over *d*_norm_.

To better understand the van der Waals inter­actions, a Hirshfeld surface analysis was performed. In Fig. 5 ▸, the Hirshfeld surface generated by *CrystalExplorer21* (Spackman *et al.*, 2021 ▸) is mapped over *d*_norm_ (Spackman & Jayatilaka, 2009 ▸) and red dots are used to represent close contacts.

**Figure 6:**
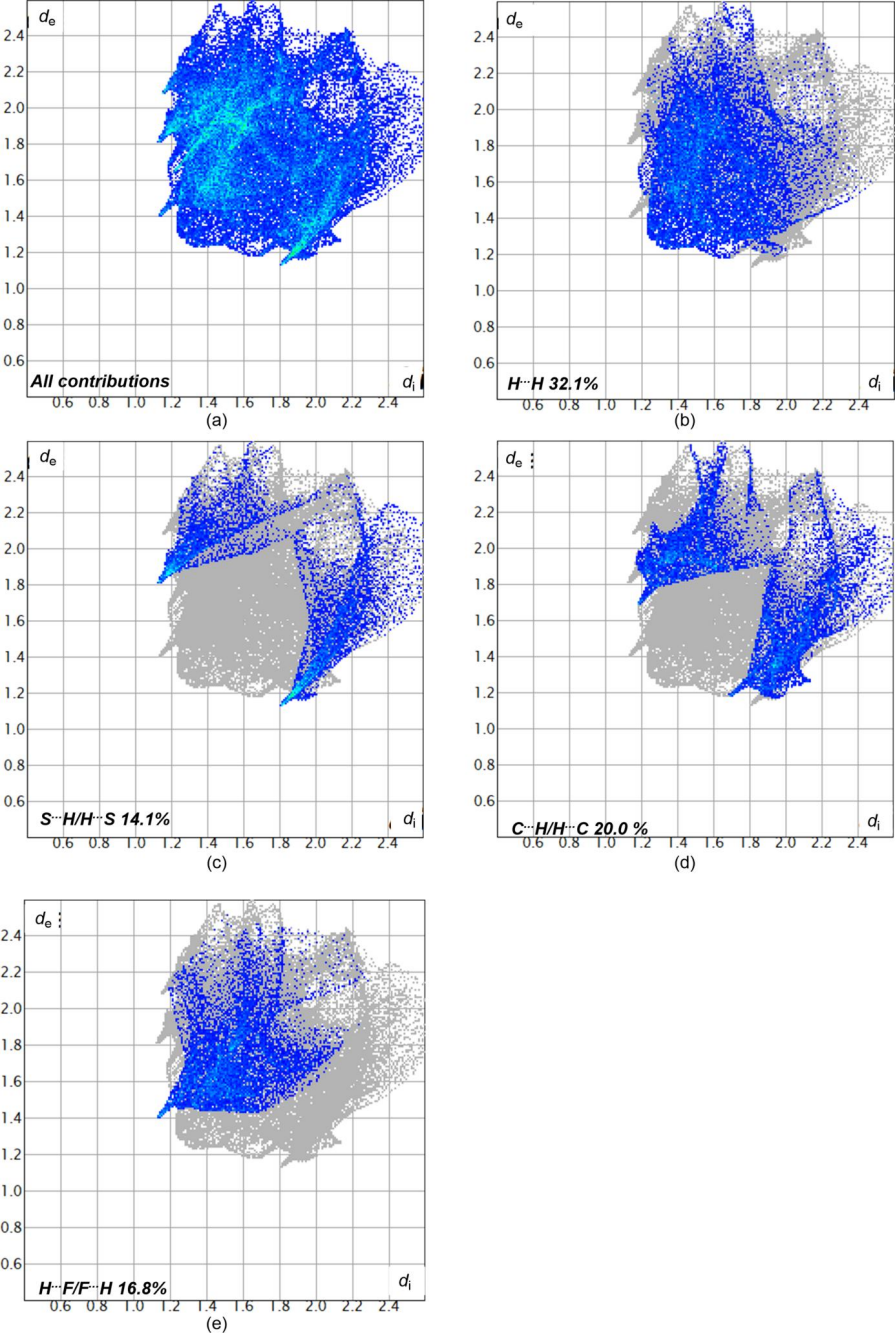
Two-dimensional fingerprint plots for **5** showing (*a*) all inter­actions, and (*b*)–(*h*) delineated into contributions from other contacts (blue areas) [*d_e_* and *d_i_* represent the distances from a point on the Hirshfeld surface to the nearest atoms outside (external) and inside (inter­nal) the surface, respectively].

For further exploration of the inter­molecular inter­actions, two-dimensional fingerprint plots (McKinnon *et al.*, 2007 ▸) were generated as shown in Fig. 6 ▸. The H⋯H inter­action with a contribution of 32.1% has the biggest impact on the packing in the solid state. The C⋯H/H⋯C bonds with 20.0%, F⋯H with 16.8%, S⋯H/H⋯S with 14.1%, N⋯H/H⋯N with 3.3%, N⋯I/I⋯N with 3.2% C⋯I/I⋯C with 2.2% or N⋯C/C⋯N with 1.5% are less impactful in comparison.

## 4. Database survey

A search of the Cambridge Structural Database (CSD, Version 5.45, last update June 2024; Groom *et al.*, 2016 ▸) for 1,2,4,5-tetra­fluoro-3,6-di­iodo­benzene yielded 802 hits. In 261 structures, the iodo atom inter­acts with a pyridine nitro­gen atom with N⋯I distances ranging from 2.662 to 3.511 Å and averaging 2.911 Å. The mean C—I⋯N angle is 171.9°.

A search for the keywords halogen bond, thio­ether and pyridine leads to a structure of 1,2,4,5-tetra­fluoro-3,6-di­iodo­benzene–4-(pyridin-4-ylsulfan­yl)pyridine (1/1) (Arman *et al.*, 2010 ▸). The variety of publications containing halogen bonds is quite large and includes the previously discussed strong halogen bond with quinuclidine (Otte *et al.*, 2023 ▸) or halogen bonds with carbonyl hypoiodites as bond donors (Yu *et al.*, 2021 ▸). The structural motif of thio­ethers is also well known, especially in the context of ligand chemistry with silicon-based thio­ethers for palladium (Schneider *et al.*, 2023 ▸; Bastero *et al.*, 2002 ▸) or silver (Nomiya *et al.*, 1996 ▸; Gaudillat *et al.*, 2023 ▸). Sulfonium-based ionic liquids (Zhao *et al.*, 2007 ▸) and other systems like (*Z*)-3-allyl-5-(4-nitro­benzyl­idene)-2-sulfanyl­idene-1,3-thia­zolidin-4-one (Moreno *et al.*, 2024 ▸) are good examples of compounds with allyl groups.

## 5. Synthesis and crystallization

Complex **5** was synthesized by adding 1,2,4,5-tetra­fluoro-3,6-di­iodo­benzene (293.96 g mol^−1^, 0.33 mmol, 0.5 eq., 97.19 mg) to a solution of 2-(allyl­thio)­pyridine (151.23 g mol^−1^, 0.66 mmol, 1.0 eq., 100,00 mg) and pentane at room temperature. The solution was stirred for one h and crystallized at 193.15 K.

^1^H NMR (400 MHz, benzene-*d*_6_, ppm): 8.24 (*dt*, *J* = 4.9, 1.4, 2H, C_5_*H*_4_N), 6.80 (*ddt*, *J* = 27.0, 6.3, 1.6, 4H, C_5_*H*_4_N), 6.41–6.34 (*m*, 2H, C_5_*H*_4_N), 5.95 (*ddd*, *J* = 16.9, 10.1, 1.5, 2H, C*H*—CH_2_), 5.15 (*dq*, *J* = 16.9, 1.5, 2H, CH—C*H*_2_), 4.93 (*dt*, *J* = 10.0, 1.3, 2H, CH—C*H*_2_), 3.85 (*dq*, *J* = 6.9, 1.3, 4H, S—C*H*_2_).

^13^C NMR (101 MHz, benzene-*d*_6_, ppm): 159.10 (*C*_5_H_4_N), 149.60 (*C*_5_H_4_N), 135.66 (*C*_6_F_4_I_2_), 134.68 (*C*_6_F_4_I_2_), 122.28 (*C*_5_H_4_N), 119.23 (*C*H-CH_2_), 117.15(*C*_5_H_4_N), 65.93 (*C*_5_H_4_N), 32.88 (CH-*C*H_2_), 15.60 (*C*_6_F_4_I_2_), 1.42 (S-*C*H_2_).

^19^F NMR (377 MHz, benzene-*d*_6_, ppm): −118.86 (C_6_*F*_4_I_2_).

## 6. Refinement

Crystal data, data collection and structure refinement details are summarized in Table 1 ▸. Hydrogen atoms were positioned geometrically (C—H = 0.95–1.00 Å) and were refined using a riding model, with *U*_iso_(H) = 1.2*U*_eq_(C) for CH_2_ and CH hydrogen atoms.

## Supplementary Material

Crystal structure: contains datablock(s) I. DOI: 10.1107/S2056989024005693/vm2304sup1.cif

Structure factors: contains datablock(s) I. DOI: 10.1107/S2056989024005693/vm2304Isup2.hkl

Supporting information file. DOI: 10.1107/S2056989024005693/vm2304Isup3.cml

CCDC reference: 2362511

Additional supporting information:  crystallographic information; 3D view; checkCIF report

## Figures and Tables

**Table 1:** Experimental details

Crystal data	
Chemical formula	2C_8_H_9_NS·C_6_F_4_I_2_
*M*_r_	704.30
Crystal system, space group	Monoclinic, *P*2_1_/*c*
Temperature (K)	100
*a*, *b*, *c* (Å)	11.184 (2), 5.2951 (6), 20.544 (3)
β (°)	96.137 (6)
*V* (Å^3^)	1209.7 (3)
*Z*	2
Radiation type	Mo *K*α
μ (mm^−1^)	2.82
Crystal size (mm)	0.25 × 0.23 × 0.07

Data collection	
Diffractometer	Bruker APEXII CCD
Absorption correction	Multi-scan (*SADABS*; Krause *et al.*, 2015 ▸)
*T*_min_, *T*_max_	0.468, 0.567
No. of measured, independent and observed [*I* > 2σ(*I*)] reflections	91740, 5719, 5342
*R*_int_	0.036
(sin θ/λ)_max_ (Å^−1^)	0.827

Refinement	
*R*[*F*^2^ > 2σ(*F*^2^)], *wR*(*F*^2^), *S*	0.019, 0.047, 1.12
No. of reflections	5719
No. of parameters	181
H-atom treatment	All H-atom parameters refined
Δρ_max_, Δρ_min_ (e Å^−3^)	0.91, −0.73

Computer programs: *APEX2* and *SAINT* (Bruker, 2016 ▸), *SHELXT* (Sheldrick, 2015*a* ▸), *SHELXL* (Sheldrick, 2015*b* ▸) and *OLEX2* (Dolomanov *et al.*, 2009 ▸).

## supplementary crystallographic information

### Bis[2-(prop-2-en-1-ylsulfanyl)pyridine] 1,2,4,5-tetrafluoro-3,6-diiodobenzene Crystal data

**Table:**

2C_8_H_9_NS·C_6_F_4_I_2_	*F*(000) = 676
*M**_r_* = 704.30	*D*_x_ = 1.934 Mg m^−^^3^
Monoclinic, *P*2_1_/*c*	Mo *K*α radiation, λ = 0.71073 Å
*a* = 11.184 (2) Å	Cell parameters from 9219 reflections
*b* = 5.2951 (6) Å	θ = 4.0–28.0°
*c* = 20.544 (3) Å	µ = 2.82 mm^−^^1^
β = 96.137 (6)°	*T* = 100 K
*V* = 1209.7 (3) Å^3^	Plate, colourless
*Z* = 2	0.25 × 0.23 × 0.07 mm

### Bis[2-(prop-2-en-1-ylsulfanyl)pyridine] 1,2,4,5-tetrafluoro-3,6-diiodobenzene Data collection

**Table:**

Bruker APEXII CCD diffractometer	5719 independent reflections
Radiation source: microfocus sealed X-ray tube, Incoatec Iµs	5342 reflections with *I* > 2σ(*I*)
Mirror optics monochromator	*R*_int_ = 0.036
Detector resolution: 7.9 pixels mm^-1^	θ_max_ = 36.0°, θ_min_ = 1.8°
ω and φ scans	*h* = −17→18
Absorption correction: multi-scan (SADABS; Krause *et al.*, 2015)	*k* = −8→8
*T*_min_ = 0.468, *T*_max_ = 0.567	*l* = −33→33
91740 measured reflections	

### Bis[2-(prop-2-en-1-ylsulfanyl)pyridine] 1,2,4,5-tetrafluoro-3,6-diiodobenzene Refinement

**Table:**

Refinement on *F*^2^	Primary atom site location: dual
Least-squares matrix: full	Hydrogen site location: difference Fourier map
*R*[*F*^2^ > 2σ(*F*^2^)] = 0.019	All H-atom parameters refined
*wR*(*F*^2^) = 0.047	*w* = 1/[σ^2^(*F*_o_^2^) + (0.0187*P*)^2^ + 0.9301*P*] where *P* = (*F*_o_^2^ + 2*F*_c_^2^)/3
*S* = 1.12	(Δ/σ)_max_ = 0.001
5719 reflections	Δρ_max_ = 0.91 e Å^−^^3^
181 parameters	Δρ_min_ = −0.73 e Å^−^^3^
0 restraints	

### Bis[2-(prop-2-en-1-ylsulfanyl)pyridine] 1,2,4,5-tetrafluoro-3,6-diiodobenzene Special details

**Table:**

Geometry. All esds (except the esd in the dihedral angle between two l.s. planes) are estimated using the full covariance matrix. The cell esds are taken into account individually in the estimation of esds in distances, angles and torsion angles; correlations between esds in cell parameters are only used when they are defined by crystal symmetry. An approximate (isotropic) treatment of cell esds is used for estimating esds involving l.s. planes.

### Bis[2-(prop-2-en-1-ylsulfanyl)pyridine] 1,2,4,5-tetrafluoro-3,6-diiodobenzene Fractional atomic coordinates and isotropic or equivalent isotropic displacement parameters (Å^2^)

**Table:**

	*x*	*y*	*z*	*U*_iso_*/*U*_eq_	
I1	0.13965 (2)	0.45457 (2)	0.60714 (2)	0.01556 (2)	
S1	0.40259 (3)	0.94040 (6)	0.63185 (2)	0.02017 (6)	
F1	−0.07102 (9)	0.05135 (19)	0.62168 (4)	0.02486 (17)	
F2	0.17962 (9)	0.28759 (19)	0.45993 (5)	0.02646 (18)	
N1	0.22969 (11)	0.8241 (2)	0.70166 (6)	0.01990 (19)	
C10	−0.03525 (12)	0.0315 (2)	0.56133 (6)	0.01663 (19)	
C5	0.34960 (12)	1.1694 (3)	0.74802 (7)	0.0211 (2)	
C7	0.17783 (15)	1.0299 (3)	0.79911 (7)	0.0245 (3)	
C11	0.09192 (11)	0.1455 (2)	0.48116 (6)	0.01691 (19)	
C9	0.05773 (11)	0.1819 (2)	0.54352 (6)	0.01517 (18)	
C6	0.27543 (14)	1.1910 (3)	0.79795 (7)	0.0248 (3)	
C4	0.32194 (11)	0.9853 (2)	0.70003 (6)	0.01659 (19)	
C8	0.15980 (14)	0.8479 (3)	0.75070 (7)	0.0236 (2)	
C1	0.49191 (14)	1.2265 (3)	0.63106 (8)	0.0259 (3)	
C2	0.53357 (17)	1.2510 (4)	0.56476 (9)	0.0324 (3)	
C3	0.64683 (17)	1.2469 (5)	0.55287 (9)	0.0376 (4)	
H3A	0.666 (3)	1.271 (6)	0.5084 (14)	0.057 (8)*	
H8	0.091 (2)	0.735 (5)	0.7506 (12)	0.037 (6)*	
H5	0.418 (2)	1.273 (5)	0.7475 (11)	0.035 (6)*	
H7	0.126 (2)	1.040 (5)	0.8317 (14)	0.042 (7)*	
H1A	0.441 (2)	1.372 (6)	0.6366 (13)	0.042 (7)*	
H2	0.463 (2)	1.259 (6)	0.5245 (13)	0.050 (8)*	
H6	0.291 (2)	1.313 (5)	0.8299 (11)	0.033 (6)*	
H1B	0.564 (2)	1.215 (5)	0.6661 (11)	0.032 (6)*	
H3B	0.714 (2)	1.232 (5)	0.5891 (12)	0.039 (7)*	

### Bis[2-(prop-2-en-1-ylsulfanyl)pyridine] 1,2,4,5-tetrafluoro-3,6-diiodobenzene Atomic displacement parameters (Å^2^)

**Table:**

	*U*^11^	*U*^22^	*U*^33^	*U*^12^	*U*^13^	*U*^23^
I1	0.01687 (4)	0.01338 (3)	0.01582 (3)	0.00101 (2)	−0.00106 (2)	−0.00098 (2)
S1	0.02284 (14)	0.01741 (13)	0.02085 (13)	0.00025 (11)	0.00504 (11)	−0.00131 (11)
F1	0.0313 (4)	0.0287 (5)	0.0160 (3)	−0.0073 (4)	0.0087 (3)	−0.0051 (3)
F2	0.0292 (4)	0.0281 (4)	0.0235 (4)	−0.0131 (4)	0.0095 (3)	−0.0036 (3)
N1	0.0244 (5)	0.0172 (5)	0.0182 (4)	−0.0040 (4)	0.0027 (4)	−0.0015 (4)
C10	0.0192 (5)	0.0171 (5)	0.0139 (4)	−0.0002 (4)	0.0031 (4)	−0.0016 (4)
C5	0.0220 (5)	0.0191 (5)	0.0217 (5)	−0.0024 (4)	−0.0004 (4)	−0.0039 (4)
C7	0.0285 (6)	0.0265 (6)	0.0192 (5)	−0.0014 (5)	0.0056 (5)	−0.0027 (5)
C11	0.0185 (5)	0.0162 (5)	0.0163 (5)	−0.0021 (4)	0.0028 (4)	−0.0003 (4)
C9	0.0166 (4)	0.0137 (4)	0.0148 (4)	0.0009 (4)	−0.0001 (3)	−0.0005 (4)
C6	0.0298 (6)	0.0234 (6)	0.0210 (6)	−0.0018 (5)	0.0019 (5)	−0.0066 (5)
C4	0.0180 (5)	0.0148 (5)	0.0165 (5)	0.0008 (4)	−0.0001 (4)	0.0002 (4)
C8	0.0269 (6)	0.0240 (6)	0.0202 (5)	−0.0068 (5)	0.0047 (4)	−0.0017 (5)
C1	0.0276 (6)	0.0232 (6)	0.0281 (7)	−0.0057 (5)	0.0082 (5)	−0.0014 (5)
C2	0.0346 (8)	0.0356 (9)	0.0274 (7)	−0.0067 (7)	0.0047 (6)	0.0066 (6)
C3	0.0336 (8)	0.0541 (12)	0.0263 (7)	−0.0108 (8)	0.0090 (6)	−0.0044 (8)

### Bis[2-(prop-2-en-1-ylsulfanyl)pyridine] 1,2,4,5-tetrafluoro-3,6-diiodobenzene Geometric parameters (Å, º)

**Table:**

I1—N1	2.8628 (12)	C7—C6	1.387 (2)
I1—C9	2.0921 (12)	C7—C8	1.384 (2)
S1—C4	1.7613 (14)	C7—H7	0.94 (3)
S1—C1	1.8158 (16)	C11—C9	1.3891 (17)
F1—C10	1.3471 (15)	C6—H6	0.92 (2)
F2—C11	1.3452 (15)	C8—H8	0.97 (3)
N1—C4	1.3422 (17)	C1—C2	1.492 (2)
N1—C8	1.3454 (18)	C1—H1A	0.97 (3)
C10—C11^i^	1.3867 (18)	C1—H1B	1.03 (2)
C10—C9	1.3895 (18)	C2—C3	1.316 (3)
C5—C6	1.391 (2)	C2—H2	1.09 (3)
C5—C4	1.3976 (18)	C3—H3A	0.97 (3)
C5—H5	0.94 (3)	C3—H3B	1.01 (2)
			
C9—I1—N1	173.90 (4)	C7—C6—C5	119.64 (13)
C4—S1—C1	102.91 (7)	C7—C6—H6	120.4 (15)
C4—N1—I1	129.30 (9)	N1—C4—S1	113.22 (10)
C4—N1—C8	117.96 (12)	N1—C4—C5	122.60 (12)
C8—N1—I1	112.43 (9)	C5—C4—S1	124.17 (10)
F1—C10—C11^i^	118.13 (11)	N1—C8—C7	123.47 (14)
F1—C10—C9	120.12 (11)	N1—C8—H8	117.6 (15)
C11^i^—C10—C9	121.74 (11)	C7—C8—H8	118.9 (15)
C6—C5—C4	118.22 (13)	S1—C1—H1A	109.2 (16)
C6—C5—H5	120.7 (15)	S1—C1—H1B	109.7 (14)
C4—C5—H5	121.0 (15)	C2—C1—S1	107.84 (12)
C6—C7—H7	122.0 (17)	C2—C1—H1A	106.4 (16)
C8—C7—C6	118.05 (14)	C2—C1—H1B	110.3 (13)
C8—C7—H7	119.9 (17)	H1A—C1—H1B	113 (2)
F2—C11—C10^i^	118.29 (11)	C1—C2—H2	115.2 (15)
F2—C11—C9	120.24 (11)	C3—C2—C1	124.68 (17)
C10^i^—C11—C9	121.45 (11)	C3—C2—H2	120.0 (15)
C10—C9—I1	121.41 (9)	C2—C3—H3A	119.0 (17)
C11—C9—I1	121.77 (9)	C2—C3—H3B	121.7 (14)
C11—C9—C10	116.81 (11)	H3A—C3—H3B	119 (2)
C5—C6—H6	120.0 (15)		
			
I1—N1—C4—S1	−4.91 (15)	C6—C5—C4—S1	177.45 (11)
I1—N1—C4—C5	174.94 (10)	C6—C5—C4—N1	−2.4 (2)
I1—N1—C8—C7	−173.81 (13)	C6—C7—C8—N1	−2.1 (2)
S1—C1—C2—C3	−117.0 (2)	C4—S1—C1—C2	−162.02 (12)
F1—C10—C9—I1	−2.97 (17)	C4—N1—C8—C7	0.3 (2)
F1—C10—C9—C11	178.18 (12)	C4—C5—C6—C7	0.5 (2)
F2—C11—C9—I1	−0.19 (17)	C8—N1—C4—S1	−177.88 (11)
F2—C11—C9—C10	178.65 (12)	C8—N1—C4—C5	2.0 (2)
C10^i^—C11—C9—I1	−178.42 (10)	C8—C7—C6—C5	1.6 (2)
C10^i^—C11—C9—C10	0.4 (2)	C1—S1—C4—N1	165.66 (10)
C11^i^—C10—C9—I1	178.43 (10)	C1—S1—C4—C5	−14.19 (14)
C11^i^—C10—C9—C11	−0.4 (2)		

Symmetry code: (i) −*x*, −*y*, −*z*+1.
